# Lost in Transcription: Frequency of Inaccurate Manually Documented Point‐of‐Care Blood Glucose and Ketone Measures in Hospital

**DOI:** 10.1002/edm2.70212

**Published:** 2026-03-31

**Authors:** Ali Hazara, Rahul D. Barmanray, Ray Wang, Mervyn Kyi, Spiros Fourlanos

**Affiliations:** ^1^ Department of Diabetes and Endocrinology The Royal Melbourne Hospital Melbourne Australia; ^2^ Department of Medicine The University of Melbourne Melbourne Australia; ^3^ Australian Centre for Accelerating Diabetes Innovations (ACADI), the University of Melbourne Melbourne Australia

**Keywords:** glucose, ketone, manual transcription, point‐of‐care

## Abstract

**Background:**

Optimal inpatient diabetes management requires accurate blood glucose (BG) and ketone (BK) documentation. In 2019, Royal Melbourne Hospital implemented hospital‐wide networked blood glucose monitoring (NBGM) (NovaBiomed StatStrip), which automatically uploads BG/BK values to point‐of‐care device software (Bioconnect). Until electronic health record (EHR) implementation in 2020, nurses manually recorded BG/BK data into paper‐records, enabling blinded gold‐standard accuracy assessment by digital software. This study evaluated the accuracy and potential clinical significance of inaccurate manual POC BG/BK values compared with NBGM records.

**Methods:**

DINGO POC, a sub‐analysis of the Diabetes IN‐hospital: Glucose and Outcomes (DINGO) study, audited paper‐based glucose charts against NBGM‐uploaded BG/BK values and BG time‐stamps to identify manual transcription inaccuracies. Discrepancy rates, magnitude and potential clinical significance were analysed.

**Results:**

4391 BG and 378 BK NBGM measures from 250 admissions over a two‐month period were assessed. Of BG measures, 325 (7.4%) were not recorded in patient charts and 558 (13%) were inaccurate. 302 (54%) inaccurate BGs had potentially clinically significant discrepancies (≥ 0.4 mmol/L). 1570 (36%) BG time‐stamps were recorded inaccurately, and 329 (7.5%) were not recorded. 524 (33%) inaccurate BG time‐stamps had discrepancies > 15 min. Of BK measures, 153 (41%) were not recorded and 18 (8%) were transcribed inaccurately. Inaccuracy rates were similar across wards and patient groups.

**Conclusion:**

Manual transcription of POC BG/BK values and time‐stamps, evaluated against blinded concurrent gold‐standard digital software, was often inaccurate with potential for clinical harm. Implementation of hospital‐wide NBGM systems with automated POC BG/BK upload to EHRs may mitigate manual transcription errors.

## Graphical abstract

Manual transcription of point‐of‐care blood glucose and ketone results into hospital charts was frequently inaccurate compared with automated networked glucose monitoring records. In a two‐month audit of 250 admissions, missing and incorrect entries were common, with many discrepancies clinically significant. Automated‐electronic upload may improve documentation accuracy and inpatient diabetes safety.

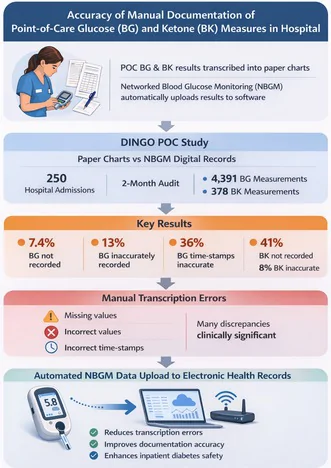

## 1 Introduction

In 2020–2021, there were almost 1.3 million hospital admissions involving people with diabetes in Australia [1]. Between 2010 and 2019, there were 78 million diabetes‐associated hospital admissions in the United States [2]. For a person with diabetes admitted to hospital, recorded point‐of‐care (POC) blood glucose (BG) and blood ketone (BK) measures inform subsequent glycaemic management interventions, necessary to prevent the well‐established adverse consequences of inpatient hyperglycaemia and hypoglycaemia [3, 4, 5]. The safety and efficacy of any such interventions rely on accurate recording of bedside POC BG and BK measurements. This is increasingly important as growing evidence demonstrates that early specialist inpatient diabetes management improves glycaemic control and clinical outcomes [6, 7].

Traditionally, POC BG measurements have been manually recorded to a paper‐based bedside glucose document or typed into the glucose chart of an electronic health record (EHR) by hospital health professionals (most commonly nurses). Manual transcription of data carries the inherent risk of recording a transcription error that has been previously reported across multiple disciplines [8, 9, 10, 11]. However, there is a paucity of research into the inaccuracy rate associated with manual transcription of BG/BK data in the clinical setting. The few retrospective studies comparing manual transcription of BG measures against automatically uploaded BG data from glucose meters, primarily in intensive care and outpatient settings, have found variable transcription inaccuracy rates ranging from 3.2% to 8.1% [12, 13, 14, 15]. However, studies evaluating inaccuracy severity or inaccuracy rates across a variety of ward types are lacking.

Although automated POC–EHR interfacing is increasingly recommended in high‐resource healthcare systems, global implementation remains heterogeneous. Even in countries with widespread EHR adoption, utilisation and interoperability vary across regions and institutions, with lower uptake in rural and resource‐limited settings. In many hospitals worldwide, manual transcription or hybrid documentation systems persist due to infrastructure, interoperability, and funding constraints [16, 17]. Despite this, quantitative real‐world data evaluating the magnitude and clinical significance of manual transcription inaccuracies in contemporary inpatient settings remain limited, particularly with respect to ketone documentation and time‐stamp discrepancies.

Networked blood glucose monitoring (NBGM) systems directly upload time‐stamped, patient identifier‐linked BG and BK measures to centralised software servers. In September 2019, the Royal Melbourne Hospital implemented an NBGM system prior to transitioning from a paper‐based medical record to a real‐time EHR in August 2020. During this interim period, BG and BK results were manually transcribed into bedside charts for clinical decision‐making, while simultaneously being automatically uploaded in real‐time to proprietary POC device software. This transition period created a unique opportunity for blinded comparison between manually recorded and digitally captured BG/BK data.

The aim of this study was to evaluate the accuracy of manual transcription of POC BG and BK values and time‐stamps compared with concurrently uploaded NBGM data, and to assess the magnitude and potential clinical significance of any discrepancies across different inpatient ward settings.

## 2 Methods

The Diabetes IN‐hospital: Glucose and Outcomes Point‐of‐Care (DINGO POC) study is a sub‐analysis of the DINGO prospective cohort study [5]. DINGO was conducted over 26 weeks between October 2019 and March 2020 at The Royal Melbourne Hospital (quaternary hospital affiliated with The University of Melbourne), Australia. The overall study design, definitions, and primary results have been previously reported. The study included patients admitted to surgical and medical wards with at least two capillary glucose measures recorded. Patients with a length of stay (LOS) < 24 h were excluded. Pre‐existing diabetes was defined as a clinical diagnosis of diabetes or pre‐hospital prescription of glucose‐lowering medications recorded at admission. For DINGO POC, admissions from October 2019 to November 2019 were included, as this two‐month period provided adequate data for this study. The study was approved by the Melbourne Health Human Research Ethics Committee (MH HREC approval number: QA2018117).

### 2.1 Data Collection

Capillary BG and BK measurements obtained using the NBGM system (StatStrip, NovaBiomedical, Waltham, Massachusetts) were automatically uploaded to proprietary POC device software on the hospital server, from which the data were extracted for analysis. All NBGM‐recorded BG/BK values and associated time‐stamps were extracted directly from the server database. The dataset was structured chronologically by individual patient and ordered by time‐stamp to replicate the layout of each patient's paper‐based glucose observation chart.

Corresponding manually transcribed BG/BK values and time‐stamps were obtained through audit of the paper glucose charts.

A manually transcribed entry was considered a match to an NBGM‐recorded result if:

1. Both the glucose/ketone value and time‐stamp were identical; or
2. Either the value or time‐stamp matched, and the result appeared in the correct chronological sequence between two confirmed matched NBGM–manual pairs.

**FIGURE 1:**
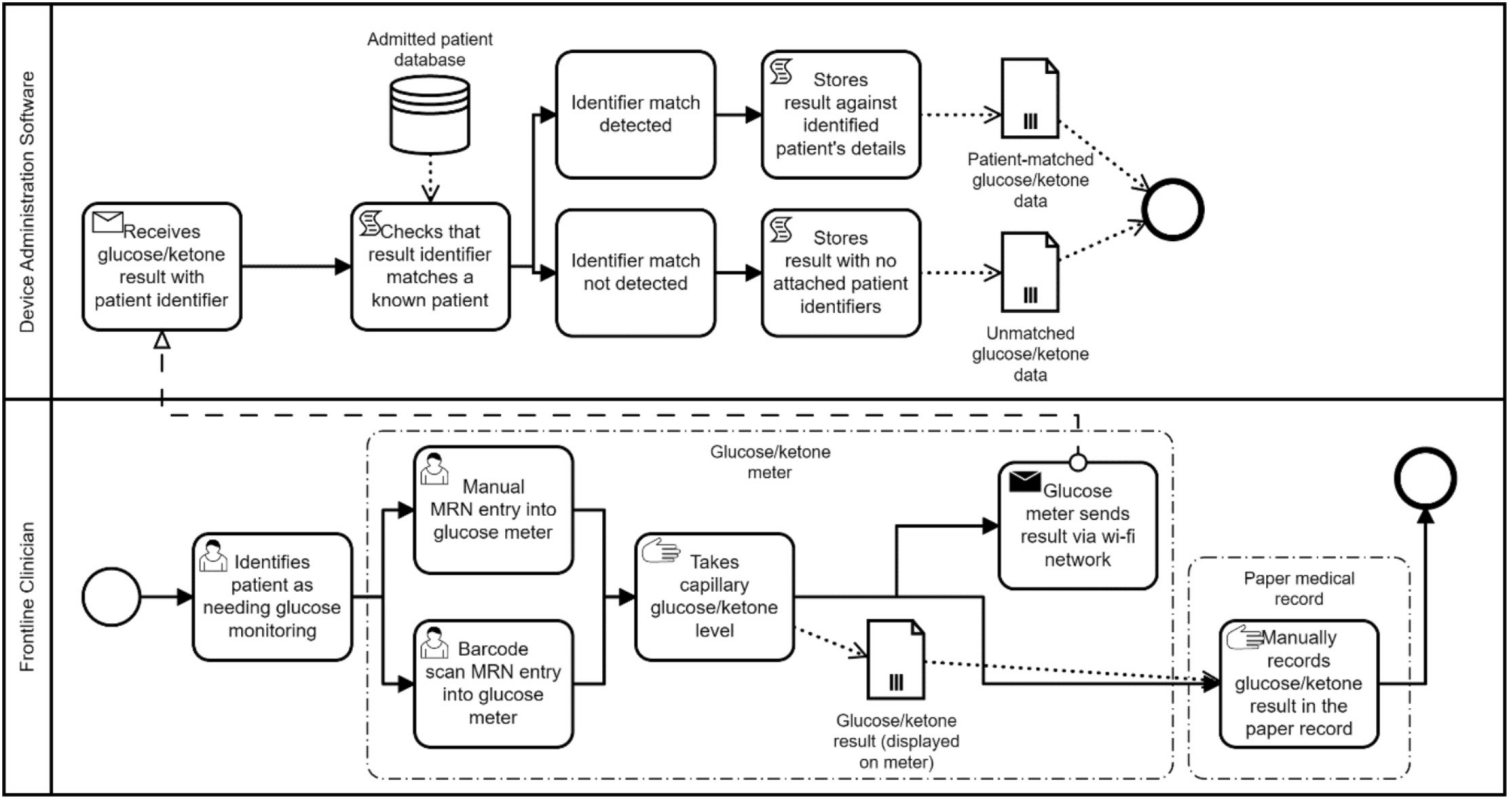
BG measure workflow in Business Process Modelling Notation (BPMN). Clinician procedures are depicted in the bottom lane, software procedures in the top lane. Hand icons indicate a manual task. Rectangular task boxes represent actions performed by clinicians or software. Document icons represent generated data outputs. Solid arrows indicate primary workflow progression; dotted arrows represent data transfer or secondary matching processes. Open circles represent workflow start or end events. Bold‐outlined circles represent terminal endpoints of matched and unmatched data pathways within the NBGM system.

Where a manually transcribed result appeared to correspond sequentially to an NBGM result but differed in both value and time‐stamp, case‐level adjudication was undertaken by a consultant endocrinologist (RDB). Determination of match status was based on review of additional contemporaneous medical record data (Figure 1).

### 2.2 Comparison of NBGM and Glucose Chart Measurements

NBGM data were compared with manually recorded entries on glucose charts for the presence or absence of matchable BG and BK results. Additionally, for BGs, any discrepancies in the BG measures and the time for BG measures were assessed. For discrepancies in BG measures, a difference of ≥ 0.4 mmol/L was considered to be a discrepancy of potential clinical significance as this is greater than the potential glucose meter inaccuracy‐related variability at the upper end of the hypoglycaemic range (with inpatient hypoglycaemia typically defined as a glucose < 4.0 mmol/L) [18].

For discrepancies in BG time‐stamps, time differences were categorised as follows: time differences ≤ 5 min were considered accurate, > 5–15 min were considered inaccurate but unlikely to be clinically significant, and > 15 min were considered potentially clinically significant, as this approximates the onset of action following subcutaneous rapid‐acting insulin administration [19]. NBGM device time‐stamps were used as the reference standard, as devices were synchronised via the hospital network to central server time and subject to routine information technology system controls. In addition, the connected glucose meters synchronise their system time and patient lists with the central software server, ensuring consistent time display across devices.

### 2.3 Statistical Analysis

Continuous variables are presented as mean ± standard deviation (SD). Categorical variables are presented as counts and percentages.

Comparisons of categorical variables between ward types (acute medical, surgical, subacute) and between patients treated with insulin therapy prior to admission versus those not treated with insulin were performed using chi‐square tests.

Omission rates between BG and BK measures were also compared using chi‐square testing.

All statistical analyses were performed using IBM SPSS Statistics version 27 (IBM Corp., Armonk, NY, USA). A two‐sided *p*‐value < 0.05 was considered statistically significant.

## 3 Results

**FIGURE 2:**
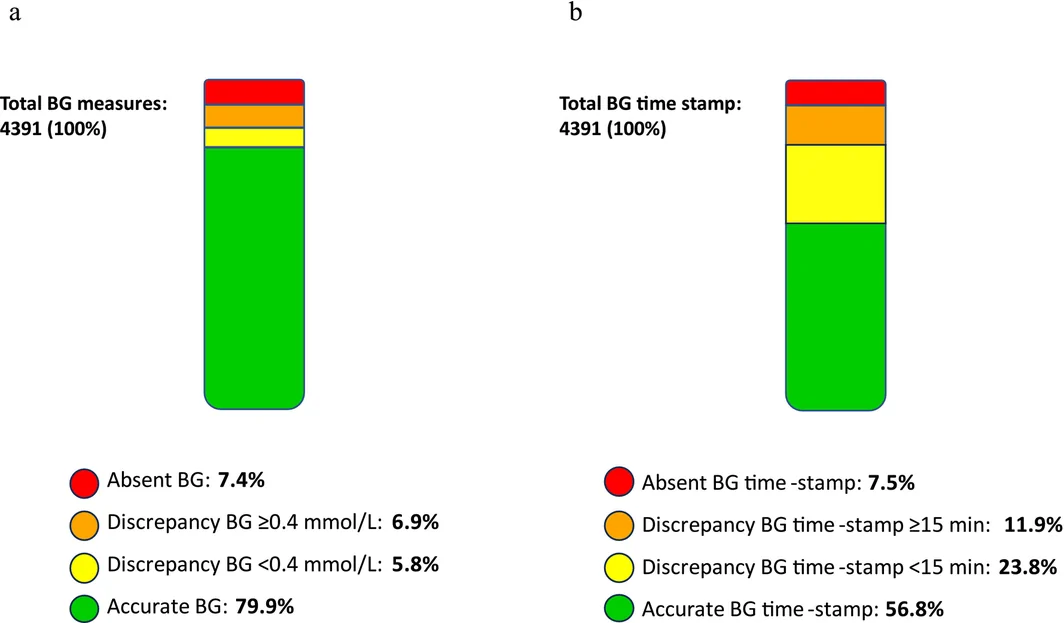
Inaccuracy frequency associated with manual transcription for (a) BG measures, (b) BG time‐stamps.

**FIGURE 3:**
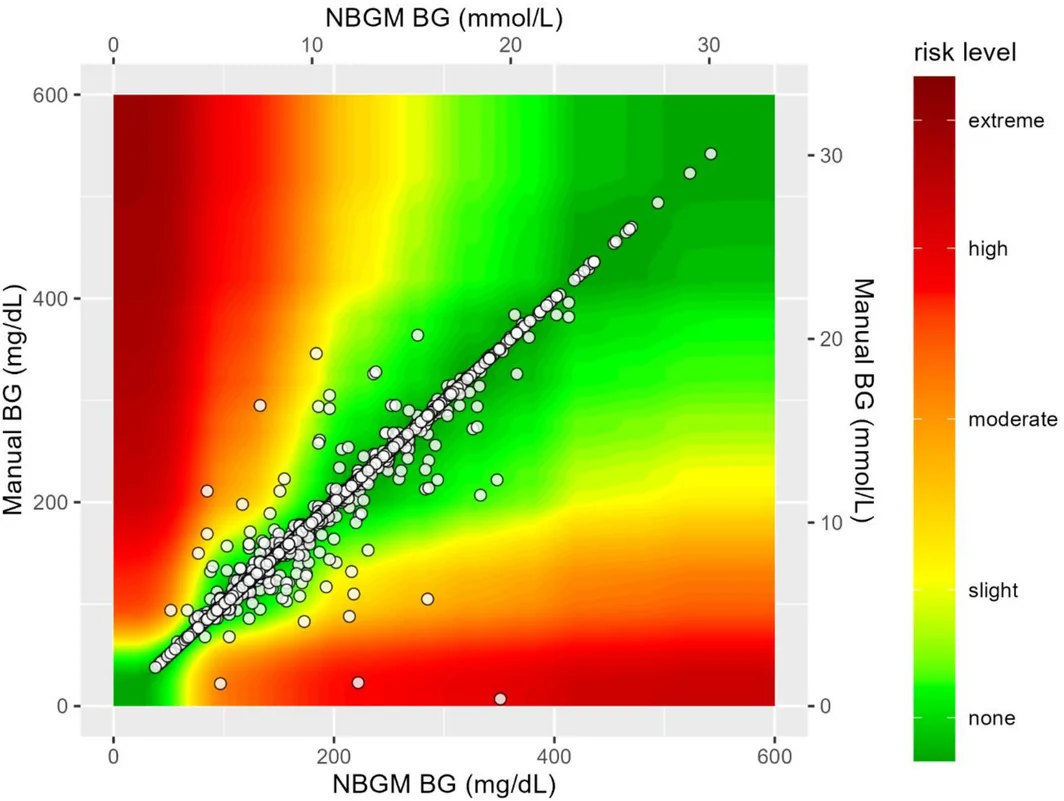
Surveillance error grid of matched NBGM and manual BG measures [20].

There were 250 admissions meeting the DINGO POC inclusion criteria, with baseline demographic and clinical characteristics summarised in Table 1, for which the NBGM system recorded a total of 4769 POC results. Of these, 4391 were BG and 378 BK measures. Compared with NBGM recorded data, 325 (7.4%) BG measures were not recorded in patient charts. There were 558 (13%) BG values that were discrepantly recorded in the medical record. Of these, 256 (46%) BG measures had a discrepancy < 0.4 mmol/L. Notably, 302 (54%) BG values had discrepancies of potential clinical significance (≥ 0.4 mmol/L) (Figures 2 and 3).

**TABLE 1:** Patient clinical characteristics.

Average age (Years)	71.1 ± 13.9
Male sex	149 (59.6%)
Modified Charlson Comorbidity Index	3.0 ± 2.1
HbA1c	
mmol/mol	56.1 ± 18.2
%	7.3 ± 1.7
Diabetes	
Diabetes treatment pre‐admission	211 (84.4%)
No medical therapy	39 (15.6%)
Non‐insulin agents	167 (79.1%)
Insulin (with or without non‐insulin agents)	84 (39.8%)
Length of stay (days)	8.3 ± 12.5
BG measurements/day	2.7 ± 1.8
BG at admission	
mmol/L	9.2 ± 4.3
mg/dL	164.7 ± 76.5
Glucocorticoid treatment during admission	31 (12.4%)
Ward type	
Acute medical	156 (62.4%)
Surgical	55 (22%)
Subacute	38 (15.2%)
Psychiatry	1 (0.4%)

For 4391 NBGM recorded BG measures with an electronic time‐stamp, 329 (7.5%) BG time‐stamps were not present in the clinical record. There were 1570 (36%) BG time‐stamps that were recorded inaccurately (> 5 min). Of these, 1046 (67%) had discrepancies ≤ 15 min. Of note, 524 (33%) BG time‐stamps had discrepancies of potential clinical significance (> 15 min) (Figure 2).

There was a total of 378 BK measures recorded by the NBGM system. Of these, 153 (41%) were not recorded in patient bedside charts. Of the 225 BK measures that were recorded, 18 (8%) were transcribed inaccurately.

### 3.1 Subgroup Analyses

The 250 admissions occurred across four ward types: acute medical (*n* = 156), surgical (*n* = 55), subacute (*n* = 38) and psychiatry (*n* = 1). Due to the single psychiatry admission, ward‐type comparison analyses were limited to acute medical, surgical and subacute wards. However, the single psychiatry admission was included in the overall analysis.

The proportion of clinically significant BG discrepancies (≥ 0.4 mmol/L) ranged from 5.1% in surgical wards to 8.1% in subacute wards (Table 2a). Missing BG documentation ranged from 6.8% (acute medical) to 8.2% (surgical). There were no statistically significant differences in rates of missing or discrepant BG values between acute medical, surgical, and subacute wards (*χ* ^2^(6) = 10.43, *p* = 0.108).

**TABLE 2:** Blood glucose measures according to (A) ward type, (B) treated with insulin therapy prior to admission^a^.

a			
Ward type			
	Acute Medical (*n* = 156)	Surgical (*n* = 55)	Subacute (*n* = 38)
	BGs = 2308	BGs = 862	BGs = 1205
Accurate BGs	1847 (80%)	699 (81%)	950 (79%)
BG Discrepancy < 0.4 mmol/L	143 (6.2%)	48 (5.6%)	63 (5.2%)
BG Discrepancy > 0.4 mmol/L	160 (6.9%)	44 (5.1%)	98 (8.1%)
Missing BGs	158 (6.8%)	71 (8.2%)	94 (7.8%)

b		
	Insulin (*n* = 84)	Non‐insulin (*n* = 166)
	BGs = 1971	BGs = 2420
Accurate BG measures	1550 (79%)	1958 (81%)
BG discrepancy < 0.4 mmol/L	128 (6.5%)	128 (5.3%)
BG discrepancy > 0.4 mmol/L	150 (7.6%)	152 (6.3%)
Missing BG measures	143 (7.3%)	182 (7.5%)

^a^ Psychiatry data (*n* = 1; 16 BGs) removed from ward comparison.

Among the 250 admissions, 84 patients were treated with insulin therapy prior to hospital admission. Rates of clinically significant BG discrepancies were 7.6% in insulin‐treated patients and 6.3% in non‐insulin‐treated patients. Missing BG documentation occurred in 7.3% and 7.5% of total BG measures, respectively (Table 2b). There were no statistically significant differences in rates of missing or discrepant BG values between patients treated with insulin therapy prior to admission and those not treated with insulin (*χ* ^2^(3) = 6.30, *p* = 0.098).

Finally, the omission rate for BK measures (41%) was significantly higher than for BG measures (7.4%) (*χ* ^2^(1) = 418.5, *p* < 0.001).

## 4 Discussion

The results from DINGO POC show that manual transcription of BG and BK measures from a POC device to a clinical health record is an error‐prone process with the potential for clinical harm. We found that 80% of BG measures and only 57% of BG time‐stamps were accurately recorded in bedside charts when compared with concurrent gold‐standard NBGM recording. In addition, 7.4% of BG measures and 7.5% of BG time‐stamps were not recorded in patient charts at all. Similar inaccuracy and omission rates of BG measures and BG time‐stamps were observed across different ward types (surgical, acute medical, subacute) and between patients prescribed insulin prior to admission and those who were not.

Previous studies have reported manual transcription inaccuracies of up to 8% of BG values, comparable to DINGO POC's finding of 13% inaccurate manual BG transcription. The DINGO POC omission rates (7.4% BG values and 7.5% BG time‐stamps) associated with manual transcription fall within the range of previously reported omission rates [13, 14, 15].

Differences in manual transcription inaccuracy rates for BG values could partially be explained by differences in study context and steps involved in documentation workflow. In an intensive care unit (ICU) study, Sowan and colleagues evaluated the transcription error rate associated with the dual process of manual transcription of BG values on a paper log and subsequent manual entry to an EHR, and reported a transcription error rate of 8.1% and an omission rate of up to 40.9% [15]. Such a high omission rate likely reflects the four‐step system of collection and reporting of BG data [15]. This high inaccuracy rate associated with multi‐step recording of BG data may have been mitigated by the one‐step automated direct transfer of BG data from the bedside device to the EHR.

Similarly, in another ICU study, Campion and colleagues reported manual BG transcription inaccuracies of up to 5.3%, compared with 12.7% in DINGO POC [12]. The lower inaccuracy rates observed in these ICU studies may reflect higher staff‐to‐patient ratios, the level of training of ICU staff, and the relatively less distractible 1:1–2 nurse‐to‐patient ratio environments in an ICU compared to acute medical or surgical wards. In an outpatient study where nursing staff manually entered BG results into an EHR, Mays and colleagues found a manual transcription inaccuracy rate of only 3.7% for BG measures [14]. The slower pace and lower acuity of outpatient clinics may facilitate more accurate manual data entry than the busy, fast‐paced, and high‐acuity wards of a quaternary hospital.

Furthermore, in a busy quaternary hospital ward that uses paper records, bedside charts are often unavailable because the patient is reviewed by multiple teams of doctors and allied health staff. As a result, nursing staff may have to make delayed retrospective chart entries. This further increases the probability of inaccurately recording BG/BK values, and particularly the time‐stamp (36% of BG time‐stamps were inaccurately recorded in our cohort).

To our knowledge, DINGO POC is the first study to report on blood ketone documentation accuracy. We found the BK measure omission rate to be nearly six times higher than for BG measures. Of a total of 378 BK measures, 153 (41%) were not recorded in patient bedside charts. It is likely that BKs were reflexively tested in the setting of hyperglycaemia and, if not elevated, not recorded in the chart. In the case of DINGO POC, 151 of 153 missing BKs were tested in the setting of intercurrent hyperglycaemia and subsequently not recorded. Subsequent analysis found that all the missing BK measures were within normal range (≤ 0.5 mmol/L), suggesting that selective documentation may have occurred when BK results were clinically abnormal.

The observations of DINGO POC in a pre‐EHR period accord with previously reported findings that post‐POC analytical inaccurate documentation can still occur if POC device BG measures are not immediately manually recorded into an EHR system at the bedside. Furthermore, manual entry of the data into the EHR system also carries an inherent risk of transcription error even in institutions where paper‐based observational charts have been replaced by EHR systems [8, 9, 10, 11, 15]. Therefore, to eliminate post‐analytical inaccuracies in BG documentation and the associated potential for adverse clinical sequelae, ideally hospitals should move towards the automatic electronic transfer of POC device clinical data, including BG/BK results, to a real‐time EHR system wherever feasible. However, accurate patient identification at the POC device, required for automated identifier matching, may itself be error‐prone and require appropriate education and system safeguards and incentivisation to address [21]. In addition, cost and system integration remain barriers to full automation of BG/BK data transfer for many hospitals. While automated POC–EHR interfacing is mandated in some healthcare systems, substantial variability in implementation persists globally due to infrastructure, interoperability, and funding constraints. Even in institutions with partial automation, manual fallback systems remain common during system transitions, outages, or device‐integration gaps [16, 17]. Therefore, the DINGO POC findings remain relevant in settings that continue to employ manual transcription systems for BG/BK measures.

The use of contemporaneously uploaded NBGM data stored in proprietary POC software (Bioconnect), which was not used for clinical decision‐making, provided a gold‐standard comparator and represents a major methodological strength of this study. In addition to quantifying overall inaccuracy rates associated with manual transcription, DINGO POC evaluated the severity of these inaccuracies. Unlike prior studies, which were largely confined to single‐ward (e.g., ICU) or outpatient settings, DINGO POC compared the inaccuracy rates across diverse inpatient wards (surgical, acute medical and subacute) and between patients prescribed insulin prior to admission and those who were not. However, the single‐centre and retrospective nature of data retrieval allowing for comparison between NBGM and manually recorded BG results limits the study especially in relation to determining clinical significance and external validity. Moreover, we did not evaluate whether insulin therapy initiated during hospital admission influenced manual transcription accuracy. It is possible that increased clinical vigilance in patients receiving inpatient‐initiated insulin may affect transcription practices, and this warrants further investigation. Furthermore, our assessment was limited specifically to the instances of missed recording or the degree of inaccurately recorded BG/BK measures and BG time‐stamps without evaluating clinical context. Hence, we were unable to identify the incidence of inaccurate BG recording leading to adverse clinical outcomes secondary to missed or inappropriate glycaemic interventions. Future studies should further explore the clinical harms arising directly from missing or erroneous BG transcription leading to inappropriate insulin‐related treatment across acute and subacute medical and surgical wards in hospital.

DINGO POC assessed the inaccuracies associated with manual transcription of BG values and time‐stamps compared with a contemporaneous hospital‐wide NBGM automated digital software system. We found that 7.4% of BG measures, 7.5% of BG time‐stamps, and 41% of BK measures were not recorded in the bedside clinical records. Furthermore, only 57% of BG time‐stamps and 80% of BG measures were accurately recorded in bedside clinical records. These manual transcription inaccuracies have the potential to directly lead to patient harm due to inadequate or excessive glycaemic treatment. However, potential clinical harms associated with manual transcription inaccuracies could be reduced with automated clinical data entry into EHRs. Our findings underscore the importance of implementing instrument‐record automated interfacing systems such as NBGM with EHRs for clinical data collection and reporting, wherever possible. Automated glucose data transcription systems for inpatient care are poised to be integral to increasing the quality of acute diabetes clinical care initiatives, glucometric benchmarking and inpatient research programs.

## Author Contributions

Ali Hazara cleaned the raw data, performed the data analysis, and wrote the early version of the draft. Rahul D. Barmanray suggested and designed the study, contributed to data analysis and assisted with manuscript preparation. Ray Wang reviewed the data, analyses, graphs and figures for accuracy. Ray Wang also created Figure 3 in the manuscript. Mervyn Kyi contributed to the manuscript preparation, critically reviewed multiple drafts and advised on figures selection in the manuscript. Spiros Fourlanos reviewed the data, analyses and presentation for accuracy, contributed to the abstract, introduction and discussion sections, and provided overall supervision and guidance throughout the process.

## Funding

The authors have nothing to report.

## Ethics Statement

The study was approved by the Melbourne Health Human Research Ethics Committee (MH HREC approval number: QA2018117).

## Conflicts of Interest

The authors declare no conflicts of interest.

## Data Availability

The data that support the findings of this study are available from the corresponding author upon reasonable request.

## References

[1] AIHW , Diabetes: Australian Facts [Web Report] [Internet]. Australian Institute of Health and Welfare; 2024, https://www.aihw.gov.au/reports/diabetes/diabetes/contents/introduction.

[2] M. Rubens , V. Ramamoorthy , A. Saxena , P. McGranaghan , and E. McCormack‐Granja , “Recent Trends in Diabetes‐Associated Hospitalizations in the United States,” Journal of Clinical Medicine 11, no. 22 (2022): 6636, 10.3390/jcm11226636. 36431114 PMC9698503

[3] G. E. Umpierrez , S. D. Isaacs , N. Bazargan , X. You , L. M. Thaler , and A. E. Kitabchi , “Hyperglycemia: An Independent Marker of In‐Hospital Mortality in Patients With Undiagnosed Diabetes,” Journal of Clinical Endocrinology and Metabolism 87, no. 3 (2002): 978–982, 10.1210/jcem.87.3.8341. 11889147

[4] A. Turchin , M. E. Matheny , M. Shubina , J. V. Scanlon , B. Greenwood , and M. L. Pendergrass , “Hypoglycemia and Clinical Outcomes in Patients With Diabetes Hospitalized in the General Ward,” Diabetes Care 32, no. 7 (2009): 1153–1157, 10.2337/dc08-2127. 19564471 PMC2699723

[5] R. D. Barmanray , M. Kyi , L. J. Worth , et al., “Hyperglycemia in Hospital: An Independent Marker of Infection, Acute Kidney Injury, and Stroke for Hospital Inpatients,” Journal of Clinical Endocrinology and Metabolism 109, no. 11 (2024): e2048–e2056, 10.1210/clinem/dgae051. 38279945

[6] R. D. Barmanray , M. Kyi , P. G. Colman , et al., “The Specialist Treatment of Inpatients: Caring for Diabetes in Surgery (STOIC‐D Surgery) Trial: A Randomized Controlled Trial of Early Intervention With an Electronic Specialist‐Led Model of Diabetes Care,” Diabetes Care 47, no. 6 (2024): 948–955, 10.2337/dc23-1905. 38237121

[7] M. Kyi , P. G. Colman , P. R. Wraight , et al., “Early Intervention for Diabetes in Medical and Surgical Inpatients Decreases Hyperglycemia and Hospital‐Acquired Infections: A Cluster Randomized Trial,” Diabetes Care 42, no. 5 (2019): 832–840, 10.2337/dc18-2342. 30923164

[8] R. Black , P. Woolman , and J. Kinsella , “Variation in the Transcription of Laboratory Data in an Intensive Care Unit,” Anaesthesia 59, no. 8 (2004): 767–769, 10.1111/j.1365-2044.2004.03834.x. 15270967

[9] M. K. H. Hong , H. H. I. Yao , J. S. Pedersen , et al., “Error Rates in a Clinical Data Repository: Lessons From the Transition to Electronic Data Transfer—A Descriptive Study,” BMJ Open 3, no. 5 (2013): e002406, 10.1136/bmjopen-2012-002406. PMC3657671 23793682

[10] S. L. Norton , A. V. Buchanan , D. L. Rossmann , R. Chakraborty , and K. M. Weiss , “Data Entry Errors in an On‐Line Operation,” Computers and Biomedical Research 14, no. 2 (1981): 179–198, 10.1016/0010-4809(81)90035-5. 7273719

[11] R. Wilton and A. J. Pennisi , “Evaluating the Accuracy of Transcribed Computer‐Stored Immunization Data,” Pediatrics 94, no. 6 Pt 1 (1994): 902–906. 7971009

[12] T. R. Campion , A. K. May , L. R. Waitman , A. Ozdas , and C. S. Gadd , “Effects of Blood Glucose Transcription Mismatches on a Computer‐Based Intensive Insulin Therapy Protocol,” Intensive Care Medicine 36, no. 9 (2010): 1566–1570, 10.1007/s00134-010-1868-7. 20352190 PMC2916042

[13] P. Carraro and M. Plebani , “Post‐Analytical Errors With Portable Glucose Meters in the Hospital Setting,” Clinica Chimica Acta 404, no. 1 (2009): 65–67, 10.1016/j.cca.2009.03.013. 19298797

[14] J. A. Mays and P. C. Mathias , “Measuring the Rate of Manual Transcription Error in Outpatient Point‐Of‐Care Testing,” Journal of the American Medical Informatics Association: JAMIA 26, no. 3 (2019): 269–272, 10.1093/jamia/ocy170. 30649499 PMC6351970

[15] A. K. Sowan , A. Vera , A. Malshe , and C. Reed , “Transcription Errors of Blood Glucose Values and Insulin Errors in an Intensive Care Unit: Secondary Data Analysis Toward Electronic Medical Record‐Glucometer Interoperability,” JMIR Medical Informatics 7, no. 1 (2019): e11873, 10.2196/11873. 30907735 PMC6452280

[16] K. C. Derecho , R. Cafino , S. L. Aquino‐Cafino , et al., “Technology Adoption of Electronic Medical Records in Developing Economies: A Systematic Review on Physicians' Perspective,” DIGITAL HEALTH 10 (2024): 20552076231224605, 10.1177/20552076231224605. 38222081 PMC10787531

[17] A. J. Anzalone , C. R. Geary , R. Dai , S. Watanabe‐Galloway , J. C. McClay , and J. R. Campbell , “Lower Electronic Health Record Adoption and Interoperability in Rural Versus Urban Physician Participants: A Cross‐Sectional Analysis From the CMS Quality Payment Program,” BMC Health Services Research 25, no. 1 (2025): 128, 10.1186/s12913-024-12168-5. 39849475 PMC11755824

[18] J. A. DuBois , R. J. Slingerland , M. Fokkert , et al., “Bedside Glucose Monitoring‐Is It Safe? A New, Regulatory‐Compliant Risk Assessment Evaluation Protocol in Critically Ill Patient Care Settings,” Critical Care Medicine 45, no. 4 (2017): 567–574, 10.1097/CCM.0000000000002252. 28169943 PMC5345889

[19] Novo Nordisk , NovoRapid & NovoMix Product Information [Product Information] [Internet] (Novo Nordisk, 2021), https://medsinfo.com.au/api/documents/NovoRapid_and_NovoMix_PI?format=pdf.

[20] D. C. Klonoff , C. Lias , R. Vigersky , et al., “The Surveillance Error Grid,” Journal of Diabetes Science and Technology 8, no. 4 (2014): 658–672, 10.1177/1932296814539589. 25562886 PMC4764212

[21] R. D. Barmanray , J. Tsan , M. Kyi , A. Gorelik , and S. Fourlanos , “The Storm That Was Delayed: The Deterioration of an In‐Hospital Diabetes Process‐Of‐Care Metric During the COVID‐19 Pandemic,” British Journal of Healthcare Management 27, no. 7 (2021): 166–171, 10.12968/bjhc.2020.0139.

